# Acknowledgement of Reviewers for 2020

**DOI:** 10.1590/2175-8239-JBN-2021-01-26

**Published:** 2021-03-26

The Editor-in-Chief and Associated Editors of the Brazilian Journal of Nephrology express their appreciation to each of the **183 individuals** who served as reviewers in 2020, contributing their time, effort and analysis of the manuscripts submitted, to ensure that only the best submitted works are published in journal.

***Reviewers***

Adriano Ammirati

Adriano Lima Neto

Alessandra Vicari

Alexandre Bignelli

Aline Antunes

Aluízio B. Carvalho

Ana Elizabeth Figueiredo

André Balbi

André Matos

André Vaz

Andrea Carla Bauer

Andrea Emília Stinghen

Andreia Watanabe

Angélica Lima

Antônio Carlos Seguro

Arjan Van Der Tol

Artur Quintiliano

Barbara Antunes

Bianca Massignan

Bruno Caldin

Bruno Eduardo Balbo

Camila Bicalho

Carla Maria Avesani

Carlos Aita

Carlos Perez Gomes

Carlos Sanchez Garcia

Carlucci Ventura

Carmen Tzanno

Carolina Moreira

Carolina Neves

Cassia Gomes 

Chris Karohl

Cibele Isaac Saad Rodrigues

Cinthia Teixeira

Claudia Oliveira

Cléo Mesa

Clotilde Garcia

Cristian Riella

Cristiane Dias

Cristiane Lara Chiloff

Cristianne Alexandre

Daltro Zunino

Daniel Calazans

Daniela Barreto

Daniela Ponce

Dayana Bitencourt

Dirceu Silva

Doğan Yücel

Domingos Otávio D'avila

Edwa Bucuvic

Elizabeth Daher

Elizete Keitel

Elvino Barros

Emerson Lima

Eric Weinhandl

Erika Rangel

Fabiana Nerbass

Felipe Tuon

Fellype Barreto

Fernando Almeida

Flávio Farias Filho

Francisco José Veronese

Francisco Salido

Gdayllon Meneses

Geraldo Silva Junior

Gino Seravalle

Giovani Gadonski

Gisele Meinerz

Hélady Sanders-Pinheiro

Helbert Lima

Hugo Abensur

Ida Maria Charpiot

Ita Heilberg

James C. Williams

Janaína Ramalho

Jocemir Ronaldo Lugon

Jochen Raimann

Jorge Paulo Matos

Jose Lopes Ramos

José Alberto Pedroso

Jose Carolino Divino Filho

Jose Lopes de Faria

José Moura-Neto

Jose Pazeli

José Suassuna

Juan Dapueto

Juliana Abrão

Juliana Ferreira

Juliana Leme

Juliana Mansur

Juliana Oliveira

Jyana Morais

Kanika Kapoor

Laila Andrade

Laila Viana

Leandro Lucca

Leandro U. Taniguchi

Leda Lotaif

Lídia Moura

Ligia Pierrotti

Lilian Palma

Luciane Deboni

Lucila Maria Valente

Lucimary Sylvestre

Lúcio Roberto Requião-Moura

Luís Andrade

Luis Gustavo Modelli de Andrade

Luís Martin

Manal Elshmaa 

Manuel Castro

Marcelino Durão Jr.

Marcelo Lopes

Marcos Sousa

Maria Aparecida Dalboni

Maria Ferraz

Maria Helena Guimarães

Maria Helena Senna

Mariana Faucz Munhoz da Cunha

Marilda Mazzali

Marina Cristelli

Maristela Bohlke

Mauricio Carvalho

Maurício Younes-Ibrahim

Maurilo Leite Júnior

Maycon Reboredo

Melani Custodio

Miguel Moysés Neto

Miguel Luís Graciano

Murilo Guedes

Murilo Neves

Natália Maria Fernandes

Nathalia Nobre Alecrim Moreira

Nilzete Bresolin

Om Mishra

Patricia Ferreira Abreu

Paula Hagemann

Paulo Rocha

Pedro Schwartzmann

Peter G. Kerr

Piotr Zapała Piotr Zapała

Polianna Lemos Moura Moreira Albuquerque

Precil Neves

Rachel Basques Caligiorne

Rafael Weissheimer

Raquel Stucchi

Regiane Cunha

Renato de Marco

Renato Foresto

Renato Watanabe

Rinaldo Mendes

Roberto Manfro

Rodrigo Campos

Rodrigo de Oliveira

Rodrigo Hagemannn

Rodrigo Neves

Rogerio da Hora Passos

Rogerio Paula

Rosilene Elias

Rui Barros

Samirah Gomes

Sebastião Ferreira Filho

Sebastjan Bevc

Sérgio Bucharles

Sergio Mezzano

Silvana Miranda

Silvia Maria Lins

Silvia Ribeiro

Stanislas Bataille

Stanley Araujo

Stéphanie Vianna

Svetoslav Nanev Slavov

Tainá Sandes-Freitas

Tarcio Braga

Thais Fernandes

Thyago Moraes

Tinus Toit

Valeria Veloso

Vanessa Banin

Vera Hermina Koch

Viktoria Woronik

Vinicius Delfino

Viviane Calice-Silva

Welder Zamoner

